# Salvage stereotactic body radiotherapy for post-prostatectomy recurrence: are we almost there?

**DOI:** 10.1093/jrr/rrag006

**Published:** 2026-02-24

**Authors:** Zhe Chen, Van Ton Tran, Masaki Matsuda, Tomoko Akita, Kan Marino, Takafumi Komiyama, Hiroshi Onishi

**Affiliations:** Department of Therapeutic Radiology, University of Yamanashi, 1110 Shimokato, Chuo City, Yamanashi Prefecture 409-3898, Japan; Radiotherapy Department, Oncology Center, Military Hospital 103, Vietnam Military Medical University, 261 Phung Hung Street, Ha Dong District, Ha Noi 12108, Vietnam; Department of Therapeutic Radiology, University of Yamanashi, 1110 Shimokato, Chuo City, Yamanashi Prefecture 409-3898, Japan; Department of Therapeutic Radiology, University of Yamanashi, 1110 Shimokato, Chuo City, Yamanashi Prefecture 409-3898, Japan; Department of Therapeutic Radiology, University of Yamanashi, 1110 Shimokato, Chuo City, Yamanashi Prefecture 409-3898, Japan; Department of Therapeutic Radiology, University of Yamanashi, 1110 Shimokato, Chuo City, Yamanashi Prefecture 409-3898, Japan; Department of Therapeutic Radiology, University of Yamanashi, 1110 Shimokato, Chuo City, Yamanashi Prefecture 409-3898, Japan

**Keywords:** prostate cancer, post-prostatectomy, biochemical recurrence, prostate bed recurrence, salvage radiotherapy, hypofractionation, stereotactic body radiotherapy (SBRT)

## Abstract

The use of hypofractionated radiotherapy (HFRT) after prostatectomy has expanded because of its radiobiological advantages and its potential to reduce treatment burden. Stereotactic body radiotherapy (SBRT), an even more condensed treatment approach, has recently emerged as a possible salvage option for men with biochemical recurrence, although its role remains investigational. To summarize current evidence, we performed a narrative review of published and ongoing studies evaluating moderate HFRT and SBRT for prostate bed salvage radiotherapy, drawing from MEDLINE, Embase, Cochrane CENTRAL, and ClinicalTrials.gov through May 2025. Across the available literature, moderate HFRT using 2.5–3.0 Gy per fraction over 15–28 fractions has shown favorable tumor control with generally acceptable safety, though some studies report increased late genitourinary toxicity with higher per-fraction doses or wide treatment margins. The phase III NRG-GU003 trial demonstrated non-inferiority of HFRT compared with conventional radiotherapy using patient-reported outcomes, supporting its clinical validity. Early SBRT experiences, often using 30–34 Gy in five fractions, have shown grade ≥ 2 genitourinary toxicity in up to approximately 29% of patients, while gastrointestinal toxicity has typically remained low. However, follow-up is short, and long-term safety remains uncertain. Although conventionally fractionated salvage radiotherapy remains the standard of care, current evidence supports moderate HFRT as an accepted alternative to conventional fractionation, whereas postoperative SBRT may be feasible in selected patients. Given limited long-term safety data and the potential for late toxicities to emerge many years after treatment, SBRT should remain investigational until results from ongoing trials become available.

## INTRODUCTION

Prostate cancer (PCa) is one of the most frequently diagnosed malignancies among men worldwide and remains a leading cause of cancer-related mortality in developed countries [1]. Radical prostatectomy (RP) is widely regarded as a definitive treatment for localized PCa, particularly in younger and otherwise healthy patients [2, 3]. However, despite its curative intent, disease recurrence remains common. In most cases, recurrence is first identified as a rising or detectable prostate-specific antigen (PSA) level, referred to as biochemical recurrence (BCR). BCR occurs in approximately 30–60% of patients, depending on the PSA threshold used and the duration of follow-up, with rates exceeding 50% in those presenting with high-risk pathological features [4–6].

In cases of BCR following RP, salvage radiotherapy (RT) offers a potentially curative option in nearly half of these patients when initiated at low PSA levels [7]. Several studies have demonstrated that postoperative radiotherapy (PORT) can eradicate microscopic residual disease and improve long-term outcomes in appropriately selected patients [8–10]. Salvage RT is currently regarded as the standard of care for patients with BCR in the post-prostatectomy (post-RP) setting and is consistently recommended by major clinical guidelines [2, 11–14]. Nonetheless, clinicians continue to face the dilemma of whether to deliver adjuvant RT immediately after surgery for high-risk patients or to defer treatment until BCR is confirmed. Evidence from the GETUG-AFU 17, RADICALS-RT, and RAVES trials with 5-year follow-up supports early salvage RT, initiated at low PSA levels, as the preferred strategy to optimize oncologic efficacy while avoiding overtreatment [15–17]. However, recent perspectives have highlighted the potential role of individualized decision-making based on PSA kinetics and pathological risk factors in refining the timing of salvage RT for selected patients [18]. RT targets the prostate bed (PB), aiming to eradicate residual disease localized to the pelvis; however, its benefit diminishes once the cancer has metastasized beyond the pelvic region, thereby limiting the potential impact of local treatment.

Conventional fractionated radiotherapy (CFRT), typically delivered as 64–72 Gy in 1.8–2.0 Gy per fraction (fr), remains the reference standard for postoperative treatment following prostatectomy. Large randomized trials, including GETUG-AFU 17, RADICALS-RT, and RAVES, have established its oncologic efficacy and late toxicity profile. Across these trials, CFRT has been associated with 5-year biochemical or event-free survival rates of approximately 85–90%, long-term freedom-from-distant-metastasis exceeding 90% in contemporary series, late grade ≥ 2 genitourinary (GU) toxicity generally below 20–30%, and clinically significant gastrointestinal (GI) toxicity below 10%, and thus serves as a well-defined benchmark for postoperative radiotherapy outcomes.

Conventionally, salvage RT is delivered over 7.5–8 weeks using standard fractionation schedules, which can be burdensome for patients and resource-intensive for healthcare systems [19]. Daily hospital visits over several weeks may cause significant physical and emotional fatigue, disrupt work and family life, and pose challenges for those living far from treatment centers. From a system perspective, extended fractionation schedules place significant demand on radiotherapy facilities, limiting capacity and contributing to treatment delays. In response to these limitations, hypofractionated radiotherapy (HFRT) has been proposed as an alternative, offering larger doses per fraction over a shorter overall treatment duration while maintaining efficacy and safety [20]. HFRT is generally classified into two categories: moderate and ultra-hypofractionated (UHF) regimens. Moderate HFRT typically delivers 2.5–3.0 Gy/fr over 15–28 fr, balancing convenience and toxicity. In contrast, UHF regimens, commonly known as stereotactic body radiotherapy (SBRT), delivers highly focused, ablative doses in as few as five fractions. Within this context, SBRT has been explored as a potential approach, employing tight treatment margins and advanced image guidance to minimize exposure to surrounding tissues [21, 22]. Its advantages include greater patient convenience, reduced healthcare burden, and potentially comparable oncologic outcomes to conventional schedules [23].

While SBRT is well established for definitive treatment of localized PCa [24], its role in the post-RP salvage setting remains investigational. With increasing interest in efficient, patient-centered treatment paradigms and growing access to advanced radiation technologies, salvage SBRT is attracting growing clinical and research attention as a potentially transformative option for PB recurrence.

This review summarizes current evidence on HFRT following RP, focusing on SBRT for PB recurrence. We first review published studies on its safety, feasibility, and early oncologic outcomes, then discuss ongoing and recently completed trials that may shape clinical practice. By integrating these data, we aim to clarify the current role and limitations of SBRT in the salvage setting, and to identify areas where further high-level evidence is required before broader clinical adoption can be considered.

## SEARCH STRATEGY AND STUDY ELIGIBILITY

To inform this narrative review, we conducted a focused literature search of MEDLINE (via PubMed), Embase, Cochrane CENTRAL, and ClinicalTrials.gov. The search targeted studies investigating salvage RT for PB recurrence, including both peer-reviewed articles published in English up to May 2025 and ongoing registered trials. Search terms included combinations of the following keywords: ‘prostate cancer,’ ‘post-prostatectomy,’ ‘biochemical recurrence,’ ‘salvage radiotherapy,’ ‘adjuvant radiotherapy,’ ‘hypofractionation,’ ‘fractionation,’ ‘stereotactic body radiotherapy,’ ‘SBRT,’ and ‘prostate bed.’ Boolean operators (‘AND’ and ‘OR’) were applied to refine the search strategy.

Inclusion criteria were as follows: studies had to examine the use of moderate HFRT or SBRT in patients with BCR following RP. Both prospective and retrospective clinical investigations were eligible, including formal clinical trials and institutional reports. Studies were selected if they reported clinical outcomes, toxicity data, dosimetric parameters, or treatment feasibility. Ongoing trials were included if their design and objectives aligned with the scope of this review.

Studies were excluded if they focused exclusively on pelvic nodal or distant metastatic disease, investigated SBRT for primary (non-postoperative) PCa, primarily evaluated the effects of androgen deprivation therapy (ADT) rather than RT.

For transparency, our initial search identified 412 records across all databases. After screening titles, abstracts, and full texts according to the eligibility criteria, 24 studies were included in this review, comprising 11 published clinical studies and 13 ongoing prospective trials.

## PUBLISHED CLINICAL EVIDENCE ON SALVAGE HFRT, INCLUDING SBRT

Most published studies on PORT have employed moderate HFRT regimens, typically delivering 2.5–3.0 Gy/fr over 4–6 weeks. These schedules aim to balance the benefits of shorter treatment duration with acceptable toxicity. Although several prospective studies have evaluated moderate HFRT and, more recently, postoperative SBRT, the majority are single-institution phase I/II trials with modest sample sizes and limited follow-up. High-level evidence remains scarce. NRG-GU003 remains the only randomized trial of postoperative hypofractionation, and there are still no randomized data evaluating postoperative SBRT. When interpreting these results, it is important to consider CFRT as the clinical reference point, since its efficacy and late toxicity profile have been defined by large randomized postoperative trials. Comparisons with CFRT provide context for understanding the potential advantages and limitations of hypofractionated approaches.

Several retrospective and early prospective studies have reported encouraging early experiences with salvage moderate HFRT targeting the PB. Kruser *et al.* [25] reported low rates of acute GU and GI toxicities (7% and 14%, respectively) in 108 patients treated with intensity-modulated RT (IMRT) or Tomotherapy® (65 Gy/26 fr), with no grade ≥ 3 GI events. Similarly, Massaccesi *et al.* [26] reported grade 2 GU toxicity in 10.2% of patients and grade 2 GI toxicity in 32.6%, with biochemical control comparable to conventional fractionation. Alongi *et al.* [27] further showed that IMRT/Tomotherapy®-based PORT significantly reduced acute GI toxicity compared to three-dimensional conformal radiotherapy, underscoring the dosimetric benefits of modern techniques.

In contrast, Cozzarini *et al.* [28] reported unexpectedly high rates of late GU toxicity (18% at 5 years) with moderate HFRT (2.35–2.9 Gy/fr). These outcomes were attributed to large treatment margins (e.g. 10 mm superiorly) as well as relatively high per-fraction doses, both established contributors to GU toxicity. Importantly, the authors also assumed an α/β ratio of 5 when converting dose equivalence for prostate cancer, which increased the total prescribed dose and substantially elevated the biologically effective dose (BED) delivered to the vesicourethral anastomosis (VUA). Consistent with this interpretation, long-term toxicity data suggest that margin reduction alone may not be sufficient to prevent severe late events. A recent single-institution analysis with 13 years of follow-up reported grade 3–5 late toxicities after hypofractionated PORT [29], despite the use of daily image guidance and a uniform 3-mm PTV margin. Taken together, these findings indicate that the cumulative BED delivered to critical structures such as the VUA may play a more significant role in long-term GU toxicity than margin size alone.

Given these observations, comparison of BED across published hypofractionated schedules provides additional insight into dose–toxicity relationships. Because Cozzarini *et al.* [28] used an α/β ratio of 5, their prescribed dose was increased, and when recalculated using the standard α/β = 3 Gy, their hypofractionated schedules correspond to BED₃ values of 114.1–135.9 Gy. The regimen reported by Ranta *et al.* [29] similarly reaches a BED₃ of 119.2 Gy. By contrast, contemporary postoperative SBRT regimens typically deliver lower BED₃ values (approximately 90–111 Gy for 30–34 Gy in five fractions). These differences imply that SBRT may not inherently pose a higher long-term GU risk, given its lower BED compared with historical hypofractionated protocols. It is also important to acknowledge that urinary function can decline years after prostatectomy even without RT [30], which contextualizes late urinary outcomes. Additional studies evaluating evolving postoperative fractionation further emphasize the need for careful dose selection in the salvage setting [31].

Prospective studies have also supported the safety of moderate HFRT. Gladwish *et al.* [32] conducted the first trial of hypofractionated PORT (51 Gy/17 fr), reporting minimal toxicity with only 6.7% grade ≥ 2 events. Lewis *et al.* [33] reported no acute grade ≥ 3 toxicities with 65 Gy in 26 fractions; actuarial late grade ≥ 3 GU toxicity reached 28% at 4 years, but only 7.1% persisted at last follow-up. Macchia *et al.* [34] reported a 5-year biochemical control rate of 86.5% with 62.5 Gy in 25 fractions, while maintaining low rates of late grade ≥ 2 GU and GI toxicity (7.3% and 1.1% at 5 years).

Additional phase II studies have further explored the feasibility of salvage moderate HFRT. A Brazilian phase II study (51 Gy/15 fr) reported acceptable toxicity, though follow-up was limited to 16 months. The PRIAMOS-1 trial evaluated HFRT (54 Gy/18 fr) in 39 patients, reporting low acute toxicity, with no grade ≥ 3 events and only mild GU and GI symptoms [35]. Collectively, these early-phase studies suggest that moderate HFRT can be delivered with acceptable short-term safety, while highlighting the limitations imposed by small sample sizes and relatively short follow-up.

More definitive evidence came from the NRG-GU003 trial, a phase III study comparing hypofractionated PORT (HYPORT) with CFRT. HYPORT was initially associated with slightly higher acute GI symptoms, which resolved within six months. At two years, HYPORT was confirmed noninferior in patient-reported GU and GI toxicities, providing level I evidence to support moderate HFRT as a more convenient alternative to conventional fractionation [36]. When interpreted relative to the well-established outcomes of CFRT, the available data suggest that moderate HFRT may achieve acceptable oncologic control with manageable toxicity in selected patients, although high-level comparative evidence remains limited.

Prospective data on postoperative SBRT are also emerging. The SCIMITAR trial, a single-arm phase II study, investigated PB SBRT (30–34 Gy/5 fr) in 100 patients. After 43 months of follow-up, late grade 2 and 3 GU toxicities were 25% and 4%, respectively; GI toxicities were both 3%. Compared with conventionally fractionated cohorts, SBRT showed no significant difference in patient-reported urinary or bowel outcomes (PROs). These findings suggest SBRT is well tolerated post-RP, though randomized data are needed to confirm long-term outcomes [37].

However, caution remains warranted when interpreting these early SBRT results because late adverse events after PORT may take a decade or more to appear. Ranta *et al.* [29] reported a median onset of 106 months for grade ≥ 3 toxicity, whereas most SBRT studies such as SCIMITAR have follow-up durations of only 2–4 years, a period during which only a small proportion of long-term events typically manifest. Accordingly, the long-term safety of postoperative SBRT remains uncertain, and extended follow-up is essential before firm conclusions can be drawn.

Key trial characteristics and outcomes are summarized in Table 1.

**Table 1:** Overview of clinical studies evaluating hypofractionated radiotherapy after prostatectomy

Author (year)	Study type (brief title)	Setting	N	Dose	ADT use	Toxicity (≥G2 GU / GI)	Biochemical control rate	Median follow-up
Kruser *et al.* [25] (2011)	Retrospective	Post-RP salvage	108	65 Gy / 26 fr (2.5 Gy/fr)	17%	Acute: 7% / 14%, Late: 15% / 4%	67% at 4 yr	32.4 mo
Alongi *et al.* [27] (2013)	Retrospective	Post-RP adjuvant/salvage	39	70 Gy / 28 fr (2.5 Gy/fr)	NR	Acute: 10% / 20%	NR	22.8 mo
Massaccesi *et al.* [26] (2013)	Phase II prospective	Post-RP adjuvant/salvage + pelvic RT	49	62.5 Gy / 25 fr (2.5 Gy/fr) + pelvic 45 Gy / 25 fr	73.4%	Acute: 10.2% / 32.6%	NR	NR
Katayama *et al.* [35] (2014)	Phase II prospective (PRIAMOS 1)	Post-RP adjuvant	39	54 Gy / 18 fr	12.8%	Acute: 0% / 17.9%	NR	NR
Cozzarini *et al.* [28] (2014)	Retrospective	Post-RP adjuvant/salvage	247	58–71.4 Gy / 20–28 fr (2.35–2.9 Gy/fr)	21%	Late: 18.1% (Grade 3+) / NR	NR	5.7 yr
Gladwish *et al.* [32] (2015)	Phase I/II prospective	Post-RP (mainly salvage)	30	51 Gy / 17 fr (3 Gy/fr)	13%	Acute: 6.7% / 0%, Late: 3.3% / 6.7%	83% at 2 yr	24 mo
Lewis *et al.* [33] (2016)	Retrospective	Post-RP salvage	56	65 Gy / 26 fr (2.5 Gy/fr)	18%	Acute: 16.1% / 3.6%, Late: 28% / 3.6% (Grade 3)	75% at 4 yr	48 mo
Macchia *et al.* [34] (2017)	Phase I/II prospective	Post-RP adjuvant/salvage + pelvic RT	124	62.5 Gy / 25 fr + pelvic 45 Gy / 25 fr	79.8%	Acute: 18.5% / 24.2%, Late: 7.3% / 1.1% (5-yr)	86.5% at 5 yr	30 mo
Buyyounouski *et al.* [36] (2024)	Phase III RCT (HYPORT/ NRG-GU003)	Post-RP adjuvant/salvage	144	62.5 Gy / 25 fr (2.5 Gy/fr)	23.6%	Late: 5% (Grade 3+) / 0%	88% at 2 yr	2.1 yr
Nikitas *et al.* [37] (2025)	Phase II prospective (SCIMITAR)	Post-RP salvage, MRI-guided SBRT	100	30–34 Gy / 5 fr (6–6.8 Gy/fr)	41%	Acute: 10% / 6%, Late: 29% / 6% (2-yr)	NR	43 mo
Ranta *et al.* [29] (2025)	Retrospective	Post-RP HYPORT	161	65 Gy / 26 fr (2.5 Gy/fr)	NR	Late: 34% (Grade 3+)	52% at 15 yr	13.5 yr

Abbreviations: ADT, androgen deprivation therapy; COPORT, conventionally fractionated postoperative radiotherapy; EQD2, equivalent dose in 2-Gy fractions; fr, fraction(s); GI, gastrointestinal; G3, grade 3 toxicity; GU, genitourinary; HYPORT, hypofractionated postoperative radiotherapy; mo, month(s); MRIgRT, magnetic resonance image-guided radiotherapy; NCCN, National Comprehensive Cancer Network; NR, not reported; NS, not significant; Post-RP, post-radical prostatectomy; RCT, randomized controlled trial; RT, radiotherapy; SBRT, stereotactic body radiotherapy; yr, year(s).

## ONGOING TRIALS: THE ROAD AHEAD

Radiation strategies for patients with BCR after RP have evolved from conventional fractionation to moderate HFRT, and more recently to UHF regimens such as SBRT. This shift is driven by advances in image guidance, improved dose delivery precision, and a growing emphasis on patient-centered care. In addition to reducing treatment burden, these regimens may offer comparable or even improved toxicity and disease control outcomes.

Several ongoing trials exemplify this trend toward shorter, more individualized regimens. Many employ five or fewer fractions at ≥5 Gy per fraction, taking advantage of the radiobiological sensitivity of PCa to higher per-fraction doses. These studies also incorporate innovations such as real-time tracking, lesion-specific boosting, and tailored ADT, aiming to maintain efficacy while enhancing convenience.

Recent multicenter prospective trials, including POPART and HYPORT-ES, have provided preliminary evidence supporting the feasibility of salvage UHF and hypofractionated PORT. Although full publications are pending, interim results presented at ESTRO 2024 are promising. In POPART, 50 patients with adverse pathological features or BCR received SBRT to the PB (32.5 Gy/5 fr, every other day). No grade ≥ 3 GI or GU toxicity occurred at 3 months; three patients experienced grade 2 GI events, and quality of life (QOL) was preserved [38]. In HYPORT-ES, 407 patients underwent postoperative IMRT (62.5 Gy/25 fr). Acute grade 2 GU and GI toxicities were 7% and 1%, respectively; grade 3 GU toxicity occurred in 1%. Late grade 2/3 GU toxicity rates were 10%/1.7%, and GI rates 1.2%/0.8%. The 2-year BCR-free survival (BCRFS) was 92%, with most patients maintaining or improving QOL [39].

Two additional phase II trials, PLUTO and EXCALIBUR, have explored distinct fractionation schedules for postoperative SBRT. PLUTO investigated once-weekly SBRT (30 Gy/5 fr) to the PB in 30 patients, with optional elective nodal irradiation (ENI, 25 Gy/5 fr) and ADT for 6–24 months. The primary endpoint was acute toxicity, with secondary outcomes including late toxicity, biochemical control, and PROs. The trial demonstrated short-term feasibility and tolerability [40]. EXCALIBUR assessed a 14-day SBRT course (alternate or consecutive days), with optional nodal irradiation and ADT. The primary endpoint was the 2-year change in GI and GU symptoms measured by the Expanded Prostate Cancer Index Composite (EPIC); secondary endpoints included long-term PROs, toxicity graded according to the Common Terminology Criteria for Adverse Events (CTCAE), and oncologic outcomes. Although not designed as a feasibility study, EXCALIBUR provides important data on PROs and toxicity profiles [41].

Two trials, esSBRT and HypoFocal SRT, illustrate precision RT strategies incorporating lesion targeting and risk adaptation. esSBRT evaluates early salvage SBRT (30 Gy/5 fr) to the PB in patients with rising PSA > 0.2 ng/ml and no metastasis. Optional ENI (25 Gy/ 5fr), focal boosts (40 Gy/ 5 fr to MRI-identified lesions), and short-term ADT are permitted. The primary endpoint is 3-year BCRFS; secondary outcomes include toxicity, survival, and PROs [42]. HypoFocal SRT applies lesion-directed SBRT (35 Gy/5 fr, alternate days) in 36 patients with isolated PB recurrence confirmed by multiparametric magnetic resonance imaging (mpMRI) and prostate-specific membrane antigen positron emission tomography/computed tomography (PSMA PET/CT). Endpoints include 2-year BCRFS (primary) and toxicity, progression, and QOL (secondary) [43]. Both trials embody a biologically individualized RT approach, aiming to enhance efficacy while limiting toxicity.

The STEREOBED trial is the first randomized phase II/III study comparing salvage SBRT with standard-of-care PORT. Patients receive SBRT (32Gy/5 fr to the PB with or without 25Gy/5 fr to whole-pelvis radiotherapy [WPRT]) or standard PORT (20–35 fr, conventional or moderate HFRT, with or without WPRT). The primary endpoint is the 2-year change in GI and GU scores on the Expanded Prostate Cancer Index Composite–26 (EPIC-26). Secondary endpoints include the International Prostate Symptom Score (IPSS), the EuroQol 5-Dimension 5-Level questionnaire (EQ-5D-5L), toxicity graded using the CTCAE, BCRFS, overall survival (OS), and metastasis-free survival (MFS) [44].

Additional feasibility studies continue to build evidence. The SHARP trial tested image-guided SBRT with advanced immobilization (31–32.5 Gy/5 fr) in 20 patients, focusing on feasibility and acute safety [45]. PLUTO-MPC, a multicenter extension of PLUTO, used the same SBRT regimen (alternate days) in 30 patients, with long-term follow-up planned [46]. The University of Michigan trial is the only randomized study directly comparing SBRT (34 Gy/5 fr) to moderate HFRT (55 Gy/20 fr), using 2-year changes in EPIC-26 urinary and bowel QOL as the primary endpoint [47].

Finally, the SUPERB trial pioneers single-fraction postoperative SBRT. Fifty patients with PSA 0.1–2.0 ng/ml, no gross recurrence or metastasis, receive 15 Gy in one session to the PB using image-guided volumetric modulated arc therapy (VMAT) with real-time motion tracking. The primary endpoint is 5-year CTCAE-defined toxicity; secondary outcomes include PROs, biochemical recurrence, and urinary, bowel, and sexual function. Although motion tracking is emphasized, the delivery platform is not specified in the protocol [48].

Taken together, these ongoing trials highlight the feasibility and ongoing clinical evaluation of hypofractionated and UHF strategies, with SBRT being increasingly adopted in the postoperative salvage setting. Despite differences in patient selection, imaging, target definition, and ADT use, their findings consistently support the safety, convenience, and oncologic potential of these approaches. Pending final results, these studies are expected to shape future standards for salvage radiotherapy after prostatectomy. Key design elements and outcomes are summarized in Table 2.

**Table 2:** Selected ongoing clinical trials of postoperative prostate bed radiotherapy

NCT number	Brief title	Status	Phase	Intervention type & dose/fractionation (dose per fraction)	Purpose / condition	Key primary outcome measures	Estimated enrollment	Estimated primary completion date
NCT03920033	SHARE	Recruiting	NA	Hypo-accel: 65.0 Gy / 26 fr (2.5 Gy/fr); Std: 66.0 Gy / 33 fr (2.0 Gy/fr)	BCR post-RP	BCRFS at 5 yr	288	Jan-2022
NCT04067570	PLUTO	Active, not recruiting	NA	SBRT: 30.0 Gy / 5 fr (6.0 Gy/fr, 1×/wk) to PB; ±25.0 Gy / 5 fr (5.0 Gy/fr) to PN; ±ADT 6–24 mo	Localized PCa eligible for post-RP RT (recurrence/adverse features)	GU/GI toxicity (acute, CTCAE v5.0)	30	Oct-2024
NCT04249154	PROMPT	Recruiting	Phase II	HypoRT: 50.0 Gy / 20 fr (2.5 Gy/fr); +short-course ADT	High-risk post-RP or salvage BCR	Acute GU/GI toxicity (PROs, CTCAE v5.0)	77	Dec-2023
NCT0431970	POPART	Recruiting	Obs.	SBRT: 31.0 Gy / 5 fr (6.2 Gy/fr) to PB; up to 32.5 Gy / 5 fr (6.5 Gy/fr) if relapse	RT toxicity; post-RP recurrence	GU/GI toxicity (acute & late; CTCAE v5.0; 60 mo follow-up)	30	Apr-2026
NCT04484038	HYPORT-EX	Recruiting	NA	HypoRT: 62.5 Gy / 25 fr (2.5 Gy/fr); IMRT + IGRT	Postop RT for PCa (adj/rescue RT for BCR)	GU/GI AEs – acute (CTCAE v5.0), late (RTOG)	300	Mar-2022
NCT04915508	EXCALIBUR	Recruiting	Phase II	IMRT-SBRT: 5 fr (every other day or consecutive ×14 d max); ±ADT	Rising PSA post-RP (adj/salv), adverse path, high-risk Decipher, or pN+	EPIC score change (GI and GU domains)	102	Aug-2026
NCT05667636	esSBRT	Recruiting	Phase II	Salvage SBRT: 30.0 Gy / 5 fr (6.0 Gy/fr) to PB; 40.0 Gy / 5 fr (8.0 Gy/fr) if macroscopic; ±25.0 Gy / 5 fr (5.0 Gy/fr) to PN; ±6 mo ADT	BCR post-RP	3-yr BFFS	103	Sep-2023
NCT05746806	HypoFocal SRT	Recruiting	Phase II	Focal SBRT: 35.0 Gy / 5 fr (7.0 Gy/fr) q2d; +6 mo ADT	Isolated PB recurrence post-RP (PSMA-PET/CT, mpMRI)	Biochemical relapse-free survival at 2 yr	36	Sep-2025
NCT06523634	STEREOBED	Recruiting	Phase II/III	SBRT: 32 Gy / 5 fr (6.4 Gy/fr) to PB ± WPRT 25 Gy / 5 fr ± 6 m ADT; SOC RT: 64–70 Gy / 32–35 fr or 52.5 Gy / 20 fr to PB ± WPRT 54–57.75 Gy / 32–35 fr or 44 Gy / 20 fr ± 6 m ADT	Persistent PSA or BCR post-RP	BCRFS at 2 yr	284	Feb-2029
NCT02976402	SHARP	Recruiting	Phase I	SBRT: 31.0 Gy / 5 fr (6.2 Gy/fr, adjuvant) or 32.5 Gy / 5 fr (6.5 Gy/fr, salvage)	Post-RP pts with adverse path or early BCR	Feasibility, CTCAE v4.0, PSA relapse, EPIC QoL	30	Nov-2022
NCT04848909	PLUTO-MPC	Active, not recruiting	Phase I	SBRT: 30.0 Gy / 5 fr (6.0 Gy/fr); nodes 25.0 Gy / 5 fr (5.0 Gy/fr, if ENI)	Post-RP pts with localized PCa	GU/GI toxicity (acute/late, CTCAE v5.0); EPIC QoL; bDFS	30	Dec-2034
NCT05038332		Recruiting	NA	SBRT: 34.0 Gy / 5 fr (6.8 Gy/fr); Nodes 25.0 Gy / 5 fr (5.0 Gy/fr); Moderate HypoFX: 55.0 Gy / 20 fr (2.75 Gy/fr); Nodes 42.0 Gy / 20 fr (2.1 Gy/fr)	Compare QoL (SBRT vs mod-hypoRT) post-RP	Toxicity (CTCAE v5.0); GI/GU QoL (EPIC-26); BCRFS; local, regional, distant failure	136	Nov-2026
NCT06941363	SUPERB	Recruiting	Obs.	SBRT: 15.0 Gy / 1 fr	BCR post-RP without visible disease	CTCAE v5.0 toxicity; QoL (EPIC-CP, ICIQ-SF, IIEF); PSA relapse	50	May-2033

Abbreviations: adj, adjuvant; AE, adverse event; ADT, androgen deprivation therapy; bDFS, biochemical disease-free survival; BCR, biochemical recurrence; BCRFS, biochemical recurrence-free survival; BFFS, biochemical failure-free survival; CTCAE, Common Terminology Criteria for Adverse Events; ENI, elective nodal irradiation; EPIC, Expanded Prostate Cancer Index Composite; EPIC-26, 26-item version of EPIC; EPIC-CP, EPIC for Clinical Practice; fr, fraction; GI, gastrointestinal; GU, genitourinary; HypoFX / HypoRT, hypofractionated radiotherapy; ICIQ-SF, International Consultation on Incontinence Questionnaire - Short Form; IIEF, International Index of Erectile Function; IGRT, image-guided radiotherapy; IMRT, intensity-modulated radiotherapy; mpMRI, multiparametric magnetic resonance imaging; NCT, National Clinical Trial; Obs., observational; PB, prostate bed; PCa, prostate cancer; PET/CT, positron emission tomography/computed tomography; PN, pelvic node; PROs, patient-reported outcomes; pN+, pathologically node-positive; QoL, quality of life; RP, radical prostatectomy; RTOG, Radiation Therapy Oncology Group; salv, salvage; SBRT, stereotactic body radiotherapy; SOC, standard of care; Std, standard; WPRT, whole pelvis radiotherapy, yr, year(s).

## KEY OUTCOME MEASURES IN REPRESENTATIVE STUDIES

Across the included studies, outcome measures were consistently categorized into four key domains: toxicity, efficacy, PROs, and treatment burden. Safety was primarily assessed through acute and late GU and GI toxicities, typically graded using CTCAE or the Radiation Therapy Oncology Group (RTOG) criteria. Efficacy endpoints commonly included BCRFS, progression-free survival (PFS), MFS, local control, OS, and cause-specific survival (CSS).

QOL was evaluated in a subset of studies using validated PRO instruments. Frequently used tools included EPIC, EQ-5D, European Organisation for Research and Treatment of Cancer Quality of Life Questionnaire Core 30 (EORTC QLQ-C30), Functional Assessment of Cancer Therapy – Prostate (FACT-P), and other symptom-specific scales assessing urinary, bowel, sexual, and overall health-related QOL. A detailed summary of PRO measures and assessment schedules is provided in Supplementary Table 1.

Only a limited number of studies incorporated treatment burden or cost-effectiveness analyses, highlighting broader considerations such as indirect costs and patient convenience in the context of value-based care.

## PATIENT SELECTION AND PRELIMINARY RECOMMENDATIONS FOR SALVAGE SBRT

While the optimal fractionation schedule for post-RP salvage RT remains under investigation, several prospective phase II studies, such as SCIMITAR, PLUTO, EXCALIBUR, and STEREOBED, have provided encouraging outcomes supporting the feasibility of SBRT. However, these data are limited by short follow-up and the absence of randomized phase III evidence. Therefore, any patient-selection framework must remain preliminary, serving only as a provisional guide while ongoing trials mature.

SBRT may be considered for investigation in carefully selected patients in whom early-phase data suggest potential suitability, such as those meeting the following criteria:

- Early biochemical recurrence (PSA ≤ 0.5 ng/ml)
- No nodal or distant metastasis confirmed by mpMRI or PSMA PET/CT
- No significant pre-existing urinary symptoms
- No prior pelvic irradiation
- Life expectancy greater than 5 years
- Availability of image-guided radiotherapy (IGRT) systems
- Ability to comply with rigorous follow-up protocols

In specific clinical scenarios, SBRT may offer logistical advantages in certain circumstances compared with moderate HFRT, including:

- Patients living in remote areas with limited access to treatment centers
- Patients with low baseline risk for GU or GI toxicity
- Facilities equipped with advanced motion management technologies
- Clinical settings where treatment efficiency or cost-effectiveness is prioritized

These considerations should not be interpreted as treatment recommendations. They reflect hypothesis-generating criteria based on limited early-phase evidence.

Until randomized comparative data become available, postoperative SBRT should be offered primarily within clinical trials or under circumstances where moderate HFRT or CFRT are not feasible.

## CLINICAL IMPLICATIONS FOR UROLOGISTS

Urologists play a central role in the postoperative management of prostate cancer, particularly in monitoring PSA levels and detecting BCR. As the first specialists to recognize rising PSA following RP, they are often responsible for initiating further evaluation and coordinating salvage RT when appropriate. Therefore, a clear understanding of evolving RT strategies is essential.

Moderate HFRT has become an accepted alternative to conventional fractionation, supported by phase III evidence demonstrating comparable efficacy and toxicity with shorter treatment duration. The emergence of SBRT has prompted investigation into even more condensed postoperative regimens. Although feasibility studies indicate that SBRT is technically achievable and generally well tolerated, these data remain preliminary. High-level evidence is still lacking, and SBRT should currently be used within clinical trials or limited to select patients after multidisciplinary discussion.

Several ongoing prospective studies are evaluating postoperative SBRT and other UHF strategies. Their results are expected to clarify whether these approaches can maintain oncologic efficacy while further reducing treatment burden. Until more mature outcomes are available, early salvage radiotherapy at low PSA levels remains the standard approach; CFRT as the clinical reference, and moderate HFRT is an evidence-supported accepted alternative.

Multidisciplinary collaboration will remain essential for optimal patient outcomes.

## CONCLUSION

While CFRT remains the standard of care for salvage treatment, moderate HFRT is now supported by phase III evidence as an accepted alternative. Postoperative SBRT may be feasible in selected patients. Nevertheless, long-term safety data remain limited, and late toxicities may emerge many years after treatment. Therefore, SBRT should remain investigational in the salvage setting until longer follow-up and results from ongoing randomized trials become available.

## CRediT authorship contribution statement

**Zhe Chen:** Writing—original draft, Investigation, Resources, Conceptualization. **Van Ton Tran:** Writing—original draft, Investigation, Resources. **Masaki Matsuda:** Writing—review & editing. **Tomoko Akita:** Writing—review & editing. **Kan Marino:** Writing—review & editing, Data Curation, Conceptualization. **Takafumi Komiyama:** Writing—review & editing, Validation. **Hiroshi Onishi:** Writing—review & editing, Validation, Supervision, Conceptualization.

## Supplementary Material

Supplementary_Table_1_rrag006
