# Supplementary material for: Salvage stereotactic body radiotherapy for post-prostatectomy recurrence: are we almost there?
Source: J Radiat Res. 2026 Feb 24;67(2):161–9. doi: 10.1093/jrr/rrag006 (PMC13019129; doi:10.1093/jrr/rrag006)
Supplement: Supplementary_Table_1_rrag006 [file supplementary_table_1_rrag006.docx]

**Supplementary Table 1. Patient-reported outcome (PRO) measures in completed and ongoing trials of postoperative prostate bed radiotherapy**

| NCT Number | Brief Title | PROM(s) used | Domains assessed | Timing of assessment |
| --- | --- | --- | --- | --- |
| NCT05746806 | HypoFocal SRT | EORTC QLQ-C30, PR25 | Overall QoL, urinary, bowel, sexual, hormonal | Baseline, 1, 3, 6, 12, 18, 24 mo |
| NCT01868386 | HypoFX | EPIC, EQ-5D, WPAI-GH | Sexual, hormonal, health utility, financial burden | Baseline, 1 yr, 2 yr |
| NCT03274687 | HYPORT | EPIC | Urinary, bowel | Baseline, end of RT, 6 mo, 1 yr, 5 yr |
| NCT03920033 | SHARE | EPIC, EORTC QLQ-C30 | Urinary, bowel, sexual, hormonal, overall QoL | Baseline, 1 wk post-RT, 6 mo, annually up to 5 yr |
| NCT04067570 | PLUTO | EPIC, EQ-5D, PORPUS-U | Urinary, bowel, health utility | Baseline through 3 yr (multiple time points) |
| NCT04249154 | PROMPT | FACT-P, IIEF-5, ICIQ-SF, IPSS, IBDQ, FACIT-TS-G | Urinary, bowel, sexual function, satisfaction | Baseline through 3 yr (annually) |
| NCT06523634 | STEREOBED | EPIC | Urinary, bowel, sexual function | Baseline, 3, 6, 12, 24 mo |
| NCT04915508 | EXCALIBUR | EPIC | GI, GU domains | Baseline, 3, 6, 12, 24 mo |
| NCT05667636 | esSBRT | FACT-P, IIEF-5, ICIQ-SF, IPSS, IBDQ, FACIT-TS-G | Urinary, bowel, sexual function, satisfaction | Baseline through 3 yr |
| NCT03541850 | SCIMITAR | EPIC-26, IPSS | Urinary incontinence, urinary obstruction, bowel, sexual function, hormone/vitality | Baseline, 1 mo, every 3 mo (1 yr), then every 6 mo (4 yr), then annually |
| NCT04848909 | PLUTO-MPC | EPIC, PORPUS-U, EQ-5D | GU, GI function; Health utility, anxiety, mobility, pain, etc. | Baseline through 6.5 yr |
| NCT05038332 | — | EPIC-26 | GU, GI domains (bowel, urinary) | Baseline, end of RT, 3, 6, 12, 24, 60 mo |
| NCT02976402 | SHARP | EPIC, CTCAE v4.0, IIEF-5 | Feasibility, PSA relapse, EPIC QoL | Baseline, 1 mo, every 3 mo (1 yr), then every 6 mo (5 yr) |
| NCT06941363 | SUPERB | EPIC-CP, ICIQ-SF, IIEF | CTCAE v5.0 toxicity; QoL; PSA relapse | Baseline, 1 mo, every 3 mo (1 yr), then every 6 mo |

Abbreviations: EORTC QLQ-C30, European Organisation for Research and Treatment of Cancer Quality of Life Questionnaire Core 30; EPIC, Expanded Prostate Cancer Index Composite; EPIC-26, 26-item short form of EPIC; EQ-5D, EuroQol five-dimension questionnaire; EQ-5D-5L, five-level version of EQ-5D; FACIT-TS-G, Functional Assessment of Chronic Illness Therapy – Treatment Satisfaction – General; FACT-P, Functional Assessment of Cancer Therapy – Prostate; GI, gastrointestinal; GU, genitourinary; IBDQ, Inflammatory Bowel Disease Questionnaire; ICIQ-SF, International Consultation on Incontinence Questionnaire – Short Form; IIEF-5, International Index of Erectile Function – 5 items; IPSS, International Prostate Symptom Score; mo, months; NCT, National Clinical Trial; PORPUS-U, Patient-Oriented Prostate Utility Scale – Urinary domain; PR25, prostate cancer-specific module of the EORTC QLQ; QoL, quality of life; RT, radiotherapy; WPAI-GH, Work Productivity and Activity Impairment Questionnaire: General Health; yr, year(s).
